# Hepatic and Extrahepatic Sources and Manifestations in Endoplasmic Reticulum Storage Diseases

**DOI:** 10.3390/ijms22115778

**Published:** 2021-05-28

**Authors:** Francesco Callea, Paola Francalanci, Isabella Giovannoni

**Affiliations:** 1 Bugando Medical Centre, Department of Molecular Histopathology, Catholic University Health Allied Sciences, Mwanza P.O. Box 1464, Tanzania; 2 Department of Pathology, Childrens’ Hospital Bambino Gesù IRCCS, 00165 Rome, Italy; isabella.giovannoni@opbg.net

**Keywords:** alpha-1-antitrypsin, fibrinogen, hepatic, extrahepatic, deficiency, manifestation

## Abstract

Alpha-1-antitrypsin (AAT) and fibrinogen are secretory acute phase reactant proteins. Circulating AAT and fibrinogen are synthesized exclusively in the liver. Mutations in the encoding genes result in conformational abnormalities of the two molecules that aggregate within the rough endoplasmic reticulum (RER) instead of being regularly exported. That results in AAT-deficiency (AATD) and in hereditary hypofibrinogenemia with hepatic storage (HHHS). The association of plasma deficiency and liver storage identifies a new group of pathologies: endoplasmic reticulum storage disease (ERSD).

AAT, the major serine protease inhibitor (Pi), is encoded by two codominant independent alleles. Heterozygous individuals (Pi MZ) synthesize two pools of the protein, the normal M fraction is entirely exported, while 85% of the abnormal Z is retained in the liver. Under conditions of clinical stimulation, the Pi MZ phenotype undergoes a unique phenomenon, characterized by an increase of synthesis of both fractions, but a divergent behavior in export. Moreover, 100% of the M fraction, but only 15% of the Z, are secreted into the blood. That results in an elevation of serum AAT levels up to the normal range associated with an increase of the amount of hepatic storage. The phenomenon has been called “Recruitment-Secretory Block” (“R-SB”) and explains why heterozygous Pi MZ individuals are not prone to develop the extrahepatic manifestations observed in Pi ZZ homozygotes as a consequence of the severe serum AAT deficiency.

HHHS occurs only in the heterozygous state. The “R-SB” phenomenon neither apply to homozygous Pi ZZ nor to heterozygous HHHS individuals, thus suggesting that the homozygosity of HHHS could be incompatible with life.

In contrast to fibrinogen that is synthesized exclusively and selectively by the liver, AAT can be synthesized by various tissues and cell types, a.o. macrophages, bile duct, pancreatic islet and sperm cells. Simultaneous accumulation of AAT in the RER of both hepatocytes and these cell types, indicates a mutation and a primary synthesis. Cells other than hepatocytes do not respond to acute phase stimuli and do not contribute to the circulating levels.

Indeed, Pi ZZ cirrhotic patients, after liver transplantation from a Pi MM donor, acquire permanently the donor’s phenotype and correct the serum AAT deficiency. Based on morphological, physiological, and serological correlations, it is suggested that AAT in cells other than hepatocyte is packaged in condensing vacuoles, lysosomes, and endocrine granules in the form of protease–antiprotease complexes and is playing an intracellular role.

The liver is the only source of fibrinogen. Therefore, the mutations causing conformational molecule abnormalities, result in exclusive hepatic storage.

The storage of mutant AAT and fibrinogen is toxic for the hepatocytes and causes chronic liver disease and cirrhosis.

Molecular analyses of the storing mutations and 3D modeling studies have localized the mutations in critical sites for correct assembly and secretion and have clarified the pathomorphogenesis of the aggregation process of the proteins within the RER.

In this article, we review clinical and experimental published data collected over 40 years. Taken together, the results appear to be of potential utility with regard to therapeutic strategies aimed to cure ERSD. In particular, they discourage the use of drugs that would increase the synthesis without a simultaneous increase of secretion. This article also addresses future research directed at small molecules that can prevent intracellular accumulation and increase the degradation of the mutant proteins.

## 1. Introduction

Endoplasmic reticulum storage diseases (ERSD) are genetic disorders affecting secretory proteins. Due to mutations in the encoding genes, the proteins may acquire an abnormal conformation of the molecule that aggregates within the rough endoplasmic reticulum (RER) instead of being regularly exported [1]. The process results in plasma deficiency and intracellular storage. The storage in turn causes chronic liver disease and cirrhosis.

The prototype of ERSD is alpha-1-antitrypsin deficiency (AATD). Its discovery represents a milestone in the medical field as it has led to clarify the pathogenesis of a subset of liver cirrhosis previously considered cryptogenic [2]. It has also clarified the pathogenesis of pulmonary emphysema in AATD [3] and that of emphysema in general [4,5].

The original recognition of AATD patients was done on the basis of identification on serum isoelectric focusing (IF) on polyacrylamide gel [6] of a variant of AAT called Z because of the slowest migration speed as compared to all other variants. The Z AAT was subsequently found to carry a point mutation Glu342Lys in the AAT gene [7]. Thus far, more than 100 polymorphisms have been identified and termed by alphabet letters from A to Z, or by the name of the borne town.

The normal variant is called M and is present in about 88% of the general population [8]. The deficient variants are classified as: (i) storing variants, the most frequent being the Pi Z (estimated incidence in European populations, around 3%, except in Sweden, where it is much higher, up to 7.6%) [9], and two rare variants (Mmalton—Phe52/53 deletion, and Siiyama—Ser52Phe). The three storing variants are characterized by aggregation of the mutant AAT in the ER of hepatocytes and by liver disease; (ii) absence of gene expression and of circulating protein (Pi Null); (iii) molecular lability and intracellular degradation (Pi S that is the second most frequent variant, representing around 7–10% in the general population) [6].

Pi S [10] and Pi Null [11] are neither susceptible to storage nor to the development of liver disease.

AAT is expressed in several tissues and cell types. However, the role, as well as the function of the protein in cells other than hepatocytes, need to be clarified.

The identification of the first mutation in the fibrinogen gamma chain gene [12] and the demonstration that the hereditary hypofibrinogenemia, in analogy with AATD, was due to the intrahepatic retention of the mutant protein [13,14,15], have led to the discovery of a new disease, hereditary hypofibrinogenemia with hepatic storage (HHHS), and to the concept of ERSD [1,15,16].

This review is an update of the immunomorphological, electron microscopic (EM), and molecular studies on AATD and HHHS. The rationale was based upon the assumption that finding a secretory protein stored in the RER of a given cell is a simultaneous indication of an underlying mutation and of a primary synthesis of the protein.

In this paper, we revised all material from our previously published clinical and experimental work carried out over the past forty years. Material, methods, patients, and ethical approval had been reported in details in the pertinent articles cited in the References.

Before reviewing the pathophysiology of the causative mutations and the morphological hallmark for diagnosis, we will first introduce some aspects of the synthesis and function of the two proteins.

In addition, we will discuss the relationship between the intracellular and extracellular AAT and fibrinogen in normal conditions and in ERSD, in view of their potential towards future strategies finalized to the treatment of ERSD.

## 2. Alpha-1-Antitryppsin Deficiency (AATD)

**Figure 1:**
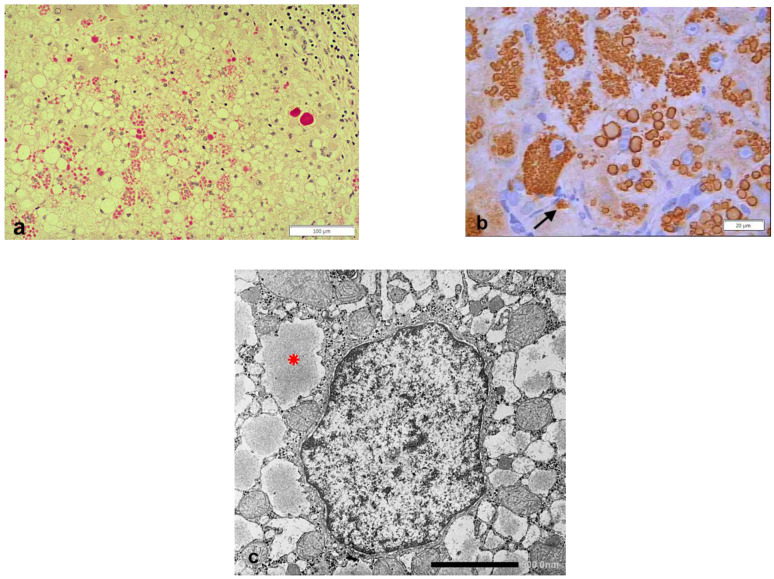
Pi ZZ liver specimen. The vast majority of hepatocytes contain PAS–D inclusions of variable size (**a**: PAS–D × 10). The inclusions are positive on immunostaining with the monoclonal AZT11 antibody (**b** × 100). The electronmicrophotograph shows a hepatocyte with dilated cisternae of the RER containing AAT-like material (*) (**c**: EM × 15,725).

According to the purposes of this review, we will summarize and discuss published data from the literature related to the variants of AAT where serum deficiency is due to the accumulation of the mutant protein variants (Z, Mmalton, and Siiyama) in the liver. The hepatocytic storage of mutant AAT presents in the form of PAS diastase (PAS–D) resistant inclusions immunoreactive to anti-AAT specific antibodies, corresponding to material in the RER (Figure 1a–c).

The molecular mechanism of Z AAT accumulation in the liver has been demonstrated as due to a loop–sheet polymerization following a molecular interaction between the reactive center loop of one molecule and the gap in the A-sheet of another [16]. The disruption of the folding is caused by the mutation Glu342Lys. Polymerization and aggregation of Mmalton and Siiyama follow a similar mechanism as their gross-mutations at positions 52/53 cause a displacement of the Beta helix that forms the base plate for the opening and closing of the A-sheet [17].

### 2.1. AAT: Source and Function

AAT is an acute phase reactant protein and the major inhibitor of serine protease (Pi) [8,18]. In addition to inhibiting a number of proteolytic enzymes [8,18], AAT plays a role in inflammation and tissue damage, coagulation, and fibrinolysis [19], immune response [20], and cell proliferation [21]. AAT is a highly polymorphic, pleiotropic, and protean protein [8,22].

The circulating AAT is synthesized by hepatocytes, exported into the blood [23,24,25,26] and is widely distributed in body fluids and interstitial tissues. The normal AAT cannot be visualized in hepatocytes because of its fast export that leaves the intracellular concentration always very low [10]. Under the EM, the normal AAT is not detectable because of its electron transparency and solubility in the ER milieu.

### 2.2. Hepatic Source and Function of AAT

Three cell types in the liver are capable of AAT synthesis: (i) hepatocytes, (ii) biliary, and (iii) Kupffer cells.

(i) Hepatocytes synthesize AAT like all secretory proteins. The AAT mRNA transcribe the message on polysomes attached to the granular endoplasmic reticulum (RER) that translates, synthesizes, and discharges the polypeptide chain into the lumen of the RER. The first sugar attachment (“core glycosylation”) occurs during translation. Then AAT translocates from the RER to the SER where further glycosylation, trimming, and elongation occur, and reaches the Golgi apparatus where terminal glycosylation takes place. Finally, the mature protein is exported into the circulation with the cooperation of microtubules [23,24,25,26].

In case of storing mutations (Z, Mmalton, Siiyama), AAT polymers aggregate within the RER cisternae and are visualized under the transmission EM (Figure 1c).

**Figure 2:**
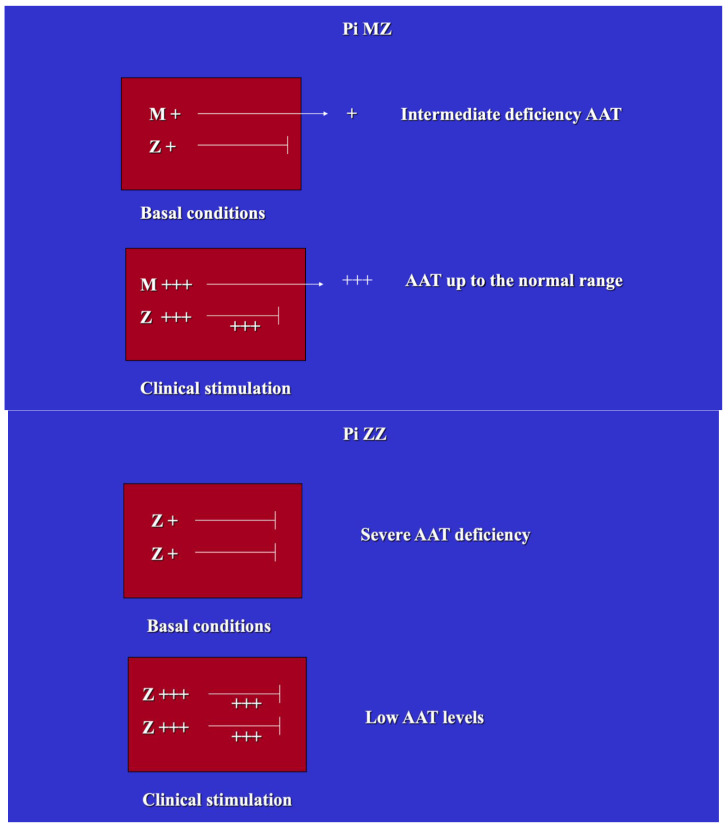
Schematic representation of the “R-SB” phenomenon in heterozygous (Pi MZ) and homozygous (Pi ZZ) AATD. Under basal conditions, Pi MZ individuals show a partial (intermediate) AAT deficiency because 85% of the Z fraction is not exported. Under conditions of clinical stimulations, the M fraction is increased in synthesis and secretion and results in serum AAT elevation. In Pi Z phenotype, the serum AAT levels are very low in both basal and stimulatory conditions because both Z fractions are retained within the cells.

AAT is encoded by two codominant independent alleles, which can occur in various combinations. The normal homozygous phenotype is designated Pi MM. The abnormal Z allele can present in a homozygous (Pi ZZ) or heterozygous (Pi MZ) state, corresponding respectively to severe or partial (intermediate) AATD. Each allele is responsible for the synthesis of 50% of the entire pool. Under conditions of clinical stimulation, Pi MZ individuals are capable of raising serum AAT levels by three–four fold, as the M fraction, due to the acute phase reactant nature of the protein, undergoes an increased synthesis and secretion rate, while 85% of the Z fraction is retained within the cell and contributes to increasing the amounts of intracellular storage. This reaction of Pi MZ to acute phase stimuli has been designated Recruitment-Secretory Block (“R-SB”) phenomenon [27]. In Pi ZZ individuals, both Z fractions are not exported, thus AAT levels remain very low (Figure 2).

**Figure 3:**
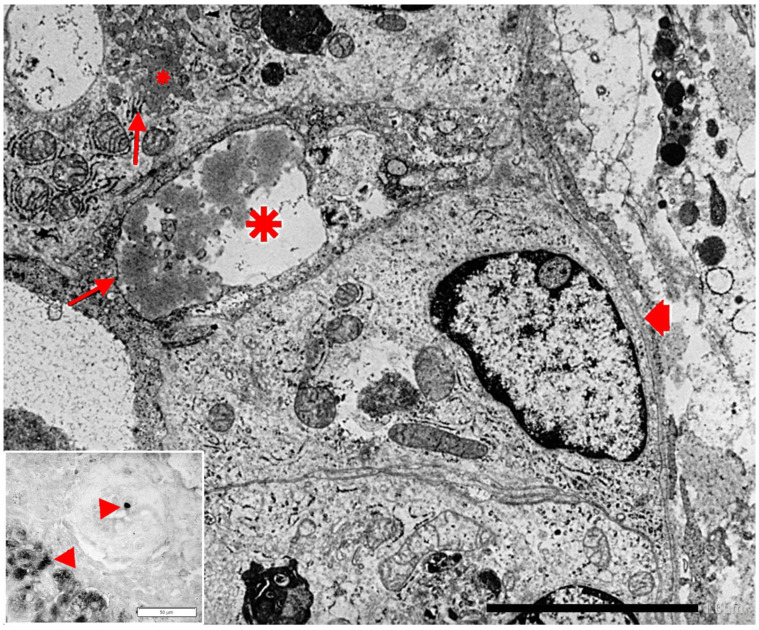
Pi ZZ patient’s liver. An interlobular bile duct shows an intercalated lining cell with an enlarged cisterna of the RER (big *) containing AAT-like material (EM × 11,500). The big arrow indicates a cytoplasmic area with communicating SER channels containing AAT-like material (small *). The inset shows the immunohistochemical staining of a tissue section from the same liver. A single large AAT inclusion is present at the apical pole of a bile duct cell (arrowhead). Periportal hepatocytes contain, as usual, AAT immunoreactive globules (arrowheads) (immunostaining × 600).

These data, together with the clinical observation that AATD (Pi ZZ) cirrhotic patients, after liver transplantation from a Pi MM donor, acquire the phenotype of the donor and normalize the serum levels [27], have definitely established that the liver is the only source of the circulating AAT [28].

(ii) Biliary cells (Hering and bile duct cells) synthesize and export AAT into the biliary system and contribute to the bile AAT pool that is estimated to represent 30% of the circulating AAT [29]. The capability of biliary cells for AAT synthesis has been discovered from studies of AATD deficient livers in which biliary cells have shown immunohistochemical and EM evidence of simultaneous accumulation of Z AAT in hepatocytes and biliary cells from the same liver [30] (Figure 3).

**Figure 4:**
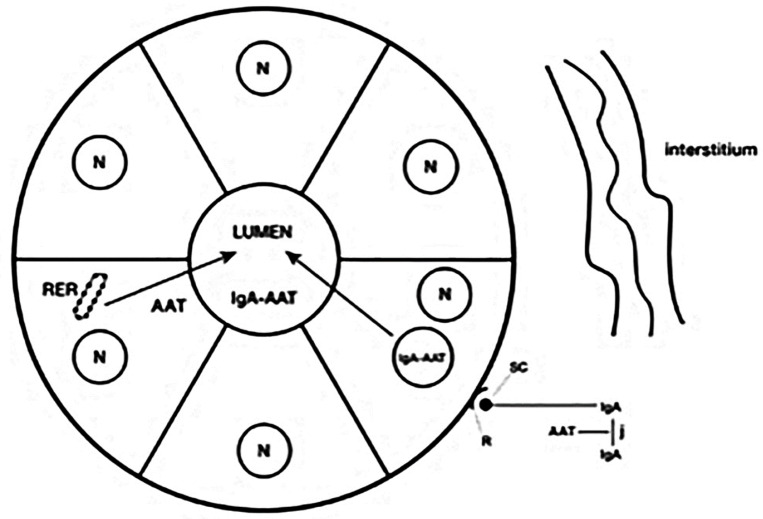
Schematic representation of transepithelial bypass of AAT bound to dimeric IgA from circulation or interstitium into the lumen of bile ducts. Mammary and salivary ducts follow the same mechanism for exporting AAT into milk and saliva. A small amount of AAT is synthesized in the RER of bile duct cells and directly secreted into the lumen.

The exact amount of AAT exported by biliary cells in not known. The bile AAT is mostly derived from transepithelial bypass of bile duct cells in human and hepatocytes in rats. Circulating dimeric IgAs as well as those derived from mucosal plasma cells, contain three ligands: J chain linking two IgA molecules, secretory component (SC) and one available to bind AAT. The SC recognizes a receptor on the basolateral membrane of epithelial cells. Subsequently, the complex (IgA/AAT/IgA/SC/J chain) is internalized via endocytosis and, through a vesicular mediated system, discharged into the lumen of bile ductules and ducts (Figure 4).

AAT bound to IgA maintains its anti-protease activity and protects the immunoglobulins from proteolysis [31].

**Figure 5:**
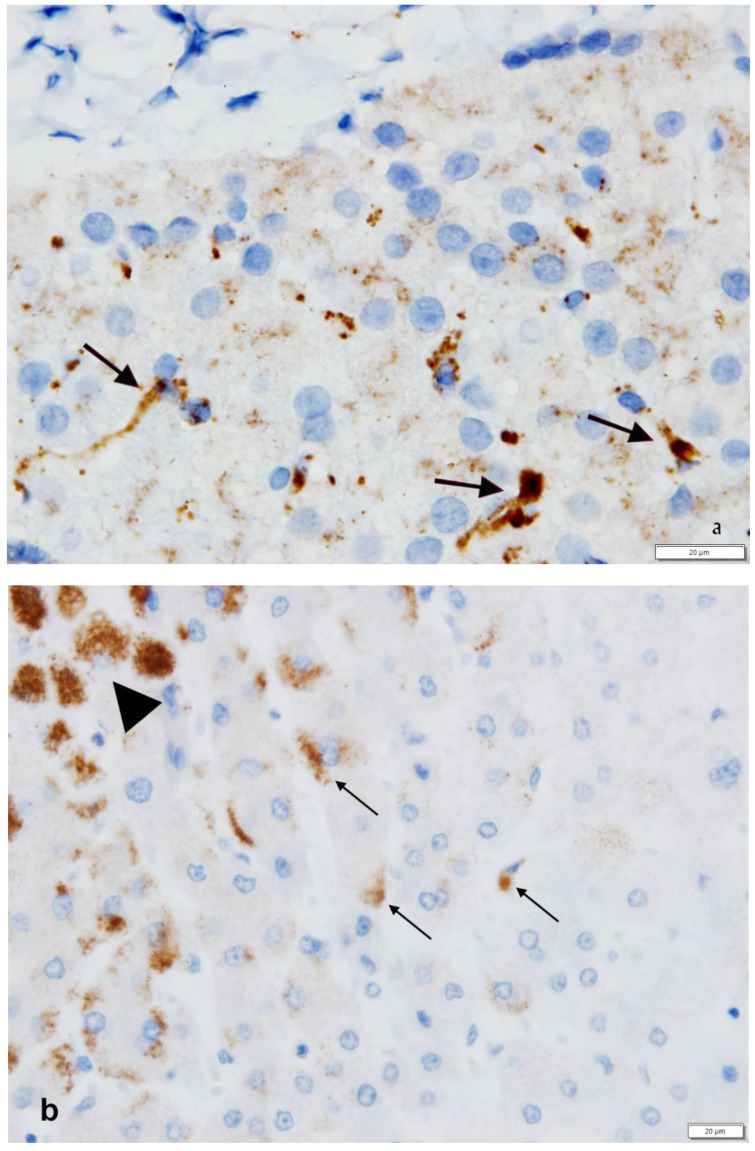
Liver tissue section from a Pi MM phenotype, stained with a polyclonal anti-AAT antibody. Hepatocytes are negative. Kupffer cells are positive. The staining appears in elongated sinusoidal lining cells in the form of granules or small globules (thin arrows), or coarse confluent globules (thick arrows) (**a**: AAT immunostaining × 100). Liver tissue section from a Pi MZ patient. AAT immunostaining is positive in both hepatocytes (arrowhead) and Kupffer cells (arrows). (**b**: AAT immunostaining × 100).

IgA and AAT play an important role in neutralizing, within the biliary system and intestine [32], bacterial, and intestinal proteases that otherwise would reach the liver via the enterohepatic circulation. The resulting endotoxemia would damage Kupffer cells and cause cholestatic liver damage. This scenario recapitulates the experimental model of galactosemia-induced cholestatic hepatitis [33], and explains the pathogenetic mechanism for neonatal cholestatic hepatitis in Pi ZZ newborns. An aggravating factor of neonatal cholestasis in Pi ZZ newborns is the AAT deficiency in the mother’s milk if they are breast fed to a Pi ZZ mother or receive only artificial milk. On the contrary, breastfed Pi ZZ newborns to a Pi MZ mother, can escape cholestasis as the mother’s serum and milk concentration is sufficiently protective, due to capability of the mother’s M fraction to raise AAT levels in blood, bile, and milk, in response to acute phase as well as to hormonal stimuli during pregnancy and lactation [10,34].

(iii) The third cell types capable of AAT synthesis in the liver are Kupffer cells. Kupffer cells are sinusoidal lining cells belonging to the monocyte/macrophage system. Like all macrophages, Kupffer cells express AAT either in normal Pi M (Figure 5a) or in Pi Z AATD deficiency (Figure 5b).

**Figure 6:**
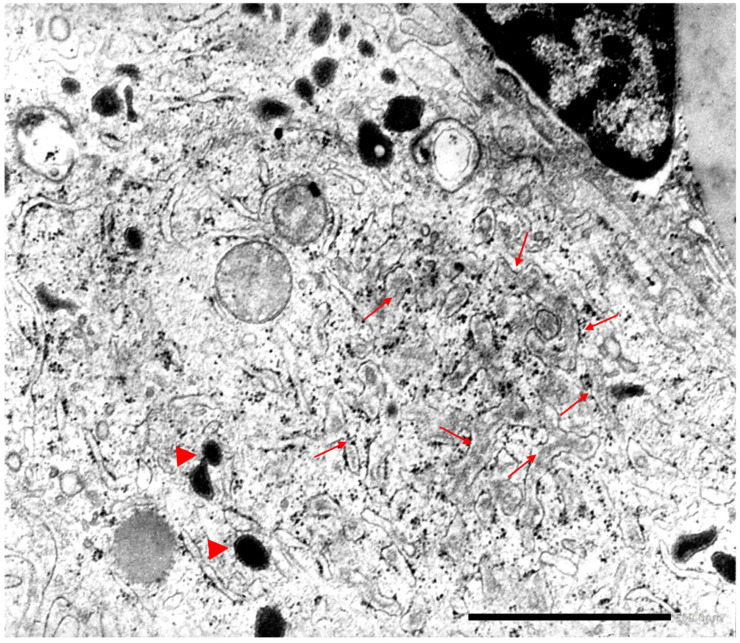
Pi MZ liver. The electronmicrophotograph shows a Kupffer cell with a large cytoplasm containing electron dense bodies (lysosomes) (arrowhead) and a rather well developed ER. The lumen of both RER and SER is slightly dilated and contains amorphous fluffy AAT-like material (arrows). A portion of a perisinusoidal (Ito) cell is present on the top right (EM × 31,025). (Figure obtained from Callea F. PhD thesis, Acco, Leuven, 1983: 1–153).

In Kupffer cells from Pi Z specimens, AAT-like material has been detected in the lumen of the RER and of the channel network of the SER, thus proving a primary synthesis and capacity of translocation in these cells (Figure 6).

However, Kupffer cells do not contribute to the circulating AAT. Therefore its presence in the ER but not in trans-Golgi SV suggests a different meaning than the hepatocytic one.

On the basis of the knowledge on protein synthesis and morphological observations, AAT could be synthesized in the RER and reach the lysosomes via the Golgi Endoplasmic Reticulum Lysosome (GERL) area, according to the “addressing organelle signal” inserted early during translation of secretory proteins [23,24,25,26], or alternatively, by free ribosomes and reach the lysosomes through the cytosol.

### 2.3. Extrahepatic Source and Function of AAT

AAT immunoreactivity has been demonstrated in monocytes/macrophages [35,36] polymorphonuclear leukocytes [37], mast cells [38], small intestinal cells [39] later on identified as Paneth cells [40], gastric mucosa [41], and pancreas endocrine cells [42].

Immunoreactivity however, per se, is not proof of primary synthesis. In this study we have assumed that finding a mutant protein within the RER of a given cell, is a proof of primary synthesis in analogy with the presence of a given protein in a monolayer medium-free cell culture, although the final proof would be the demonstration of functioning mRNA in that cell.

Gene expression of AAT has been demonstrated in human monocytes/macrophages in both Pi MM normal phenotypes and AATD [43,44]. Therefore, our finding of ZAAT in both RER and SER of AATD patients as shown in Figure 6, is in keeping with of the above molecular analysis results.

**Figure 7:**
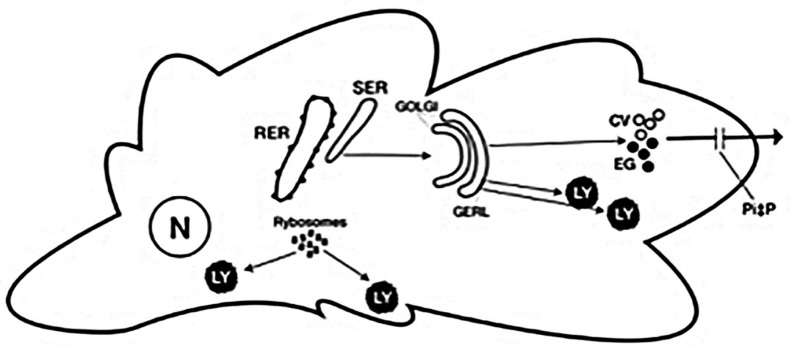
Schematic representation of the possible intracellular pathways of AAT in macrophages, polymorphonuclear leucocytes, mast cells, Paneth cells, endocrine cells. AAT synthesized in the RER reaches the Golgi apparatus and is stocked within condensing vacuoles (empty circle) or endocrine granules (black dots) or lysosomes (Ly). In the GERL area AAT recognizes specific endomembrane receptors and is diverted to lysosomes. In condensing vacuoles, endocrine granules, AAT is supposed to bind protease (Pi/P complex). After AAT cleavage (gate), proteases get activation. AAT synthesized by free ribosomes can be addressed directly to lysosomes (Ly). The dark color of endocrine granules and lysosomes reflects the electrondensity of their matrix.

Our EM observations have shown that Kupffer cells have not only the capability of AAT primary synthesis but also of translocating the protein to the SER. As Kupffer cells are not exporting AAT into the circulation, one can reasonably assume that they use the protein for intracellular purposes. At the level of the GERL area, AAT would recognize an endomembrane specific receptor and would be diverted to lysosomes, according to the organelle addressing signal incorporated at the time of the early synthesis [23,24]. Inside the lysosomes, the protein would bind proteolytic enzymes to form protease/anti-protease (P/Pi) complexes that would subsequently be discharged by exocytosis outside the cell after AAT cleavage (Figure 7).

AAT in polymorphonuclear leukocytes, mast cells, Paneth cells containing large amounts of proteolytic enzymes, fulfills intracellular requirements, namely protection against proteolytic self-digestion, and discharge the uncoupled P and Pi into the extracellular/interstitial matrix, after Pi cleavage.

The same pathway and function could be shared by endocrine cells. Once incorporated into endocrine granules (equivalent of condensing vacuoles or lysosomes) at the GERL level, AAT would temporarily form Pi/P complexes and inhibit the proteases that would activate pro-hormones into hormones, after AAT cleavage (Figure 7) [45].

**Figure 8:**
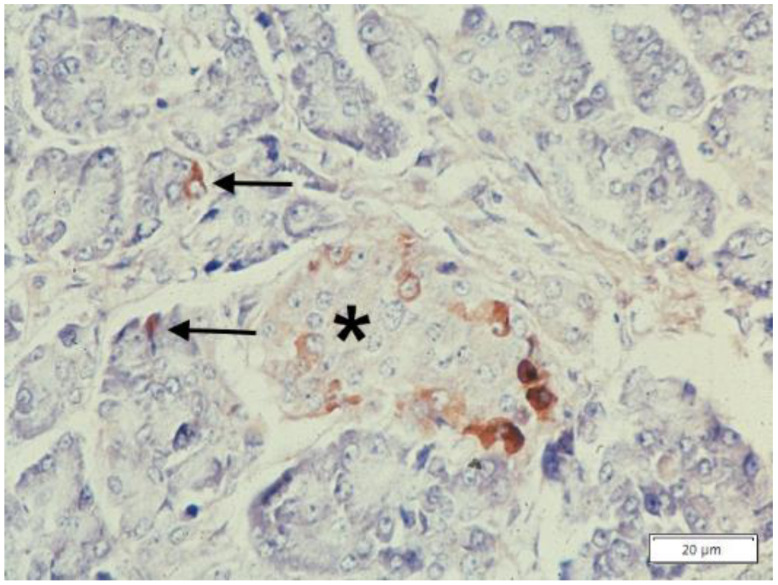
Histological section from a Pi MM phenotype pancreas stained with a polyclonal anti AAT antibody. An islet (*) contains AAT positive cells mainly located at the periphery. A few AAT positive paracrine cells are scattered in the exocrine pancreas acini (arrows) (AAT immunostaining × 100).

**Figure 9:**
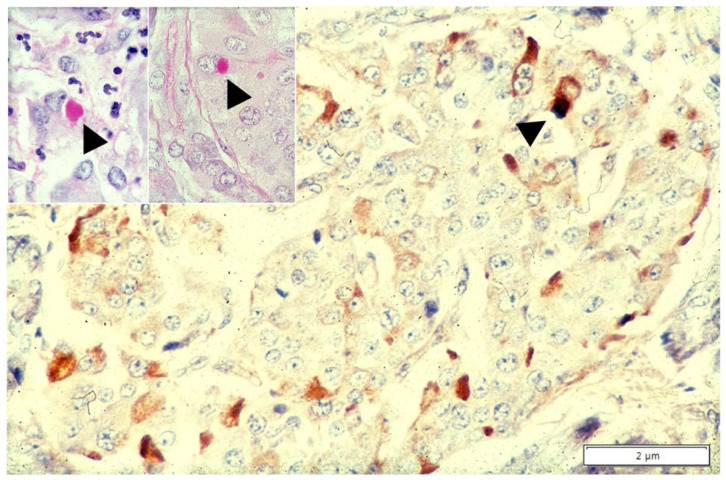
Pi ZZ cirrhotic patient. A pancreatic islet shows AAT positive cells mainly located at the periphery (immunostaining × 100). An islet cell (head arrow) contains a large AAT immunoreactive globule (arrow-head) corresponding to the PAS–D inclusion at the apical pole of the same cell (inset: arrowhead, PAS–D × 100)). An analogous inclusion is present in a hepatocyte surrounded by inflammatory cells, from the cirrhotic liver of the same patient (PAS–D, original magnification 100×).

To synthesize, the consistently AAT positive staining in islet and paracrine pancreas (Figure 8) and in Kuppfer cells (Figure 5b) of Pi MM individuals strongly supports the interpretation that AAT is not a protein for secretion in those cells (44). On the other hand, the simultaneous accumulation of Z AAT in hepatocytes, pancreatic islet (Figure 9) and Kupffer cells (Figure 6), indicates a primary synthesis in all these cells.

On the other hand, the consistently M AAT positive staining in islet and paracrine pancreas (Figure 8) and in Kupffer cells (Figure 5b) of Pi MM individuals, strongly supports the interpretation that AAT is not a protein for secretion in those cells [45].

**Figure 10:**
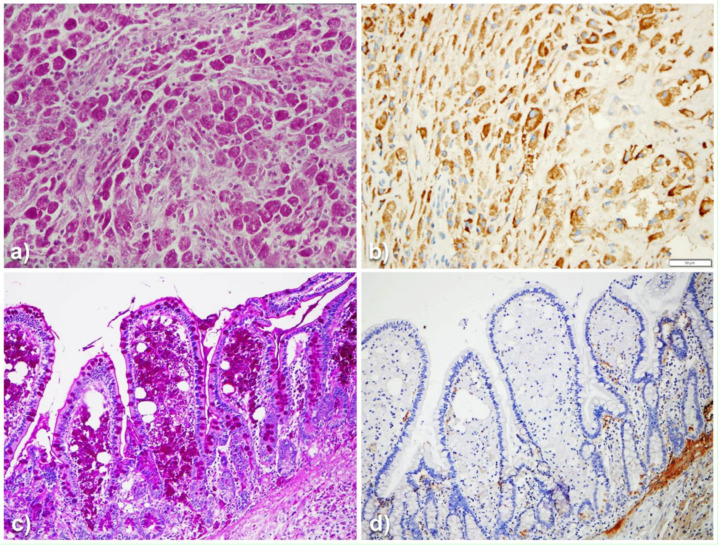
Bladder malakoplakia (**a**,**b**). Plagues of PAS–D positive macrophages (**a**: PAS–D × 40) are strongly positive for AAT (**b**: AAT immunostaining × 20). Whipple disease (**c**,**d**). Intestinal villi contain PAS–D positive macrophages (**c**: PAS–D × 40). Goblet epithelial cells and glycocalyx are also positive on PAS–D. Whipple macrophages are negative on AAT immunostaining (**d**: AAT immunostaining × 60).

In this article, we provided new findings on the neglected role of AAT in cells other than hepatocytes. The direct transfer of morphological findings into physiological or pathological interpretations, requires always caution. That holds true especially if the observations are new. A topical example is the different behavior of AAT in macrophages of malakoplakia and Whipple’s disease, two diseases of the monocyte/macrophage system that apparently share the pathomorphogenesis. Malakoplakia is a granulomatous histiocytic lesion mainly occurring in the urinary tract, resulting from a massive proliferation of von Hansemann macrophages [46]. The cytoplasm of these macrophages is filled up with PAS–D inclusions (Figure 10a) without or with calcifications (so called Michaelis–Gutmann bodies), positively stained by anti-AAT antibodies (Figure 10b).

Under the EM, malakoplakia macrophages are flooded by lysosomes and contain intact non-membrane bound bacteria [46,47], especially *E. coli*. These findings concur to point out a lysosomal loss of function possibly due to the lack of P/Pi complexes dissociation [48]. In Whipple’s disease, the lamina propria of the small intestine is obscured by large amounts of macrophages (Figure 10c) that look like malakoplakia macrophages and contain free-living bacilli referred to as Tropheryma whippelii [47,48,49]. Whipple macrophages; however, in contrast to malakoplakia macrophages, do not show AAT positivity (Figure 10d), thus suggesting a different pathomorphogenesis [48,49,50]. AAT immunostaining with polyclonal antibodies remains the hallmark for the diagnosis of malakoplakia [46].

In synthesis, we have provided evidence for a simultaneous accumulation of Z AAT in hepatocytes, Kupffer and endocrine pancreatic cells. Further, we have shown that Kupffer and pancreatic endocrine cells synthesize M AAT in normal Pi MM individuals but do nor secrete it in the blood. In those cells, the protein is concentrated within electron dense organelles (Figure 7), is detected by specific antibodies, but not visualized under the EM because it is masked by the electron density of the organelle matrix. The M AAT is not visualized neither in light microscopy due to its low intracellular concentration, or under the EM because of its solubility in the ER milieu and its intrinsic electron translucency.

**Figure 11:**
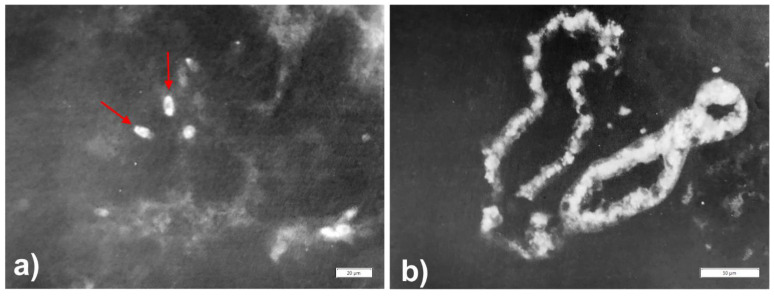
Immunofluorescence staining for AAT on an autopsy testis (**a**) and kidney (**b**) specimen from a Pi Z patient. A few spermatozoa are seen in the lumen of the epididymal duct. Heads of sperm cells shows AAT immunoreactive dots. The immunoreactive dots correspond to the acrosome (arrows) (**a** × 516). Immunofluorescence staining for AAT on a kidney from the same patient. Two proximal tubules show a very strong granular–globular positivity for AAT (**b** × 320). (Figures reprinted from Callea F. PhD thesis. Acco, Leuven 1983: 1.153).

For the sake of completeness, two additional cellular locations of AAT should be mentioned: sperm cells and proximal renal tubules. In sperm cells, AAT immunoreactivity depicts the acrosomal cup [10], most probably coupled with proteolytic enzymes, mainly acrosin [51]. The AAT staining pattern in sperm cells, indeed, reproduces the morphology of the acrosome (Figure 11a). This structure envelops the head of sperm cells in the form of a Phrygian hat. The acrosome is a highly specialized structure resulting from the coalescence of pro-acrosomal granules, which originate from the Golgi complex [52]. After AAT cleavage and P–Pi dissociation, acrosin would be activated and allow sperm cell capacitation and fertilization [10,53].

AAT immunoreactivity has been demonstrated in the kidney of both rats [54] and human (10]. However, the primary synthesis of AAT in proximal renal tubules has not been proven. AAT immunoreactive inclusions can be observed in any kidney tubular cells, regardless of the Pi phenotype. Therefore, a reabsorption process for degradation could be the explanation even when the phenomenon occurs in AATD individuals as we found (Figure 11b) [10].

### 2.4. Hepatic Manifestations in AATD

Two codominant alleles encode the synthesis of AAT; therefore, the deficiency can present either in heterozygous (Pi MZ) or homozygous (Pi ZZ) condition. The two other deficient variants, Mmalton and Siiyama, share the same behavior. In histological sections, the stored AAT appears in the form of round eosinophilic cytoplasmic inclusion bodies that are strongly positive on PAS–D stain (Figure 1a) due to the high carbohydrate content. Confirmation of the nature of the inclusions is obtained with polyclonal [55] or monoclonal [56] anti-ZAAT antibodies (Figure 1b). The discriminating immunomorphological and EM features between the three forms have been described in previous work [57,58]. Based upon epidemiological studies, the storage process per se has been considered for long time insufficient to cause liver disease and endogenous or exogenous co-factors have been searched for.

Nowadays there are no more doubts about the intrinsic toxic capacity of the stored AAT [16,58,59]. Although AATD individuals, either hetero- or homo-zygotes, may not show clinical or biochemical signs of liver disease, all of them undergo accumulation of the mutant protein Therefore, the storage process represents the true elementary lesion of the disease and reflects the phenotype-genotype correlation [16,58].

**Figure 12:**
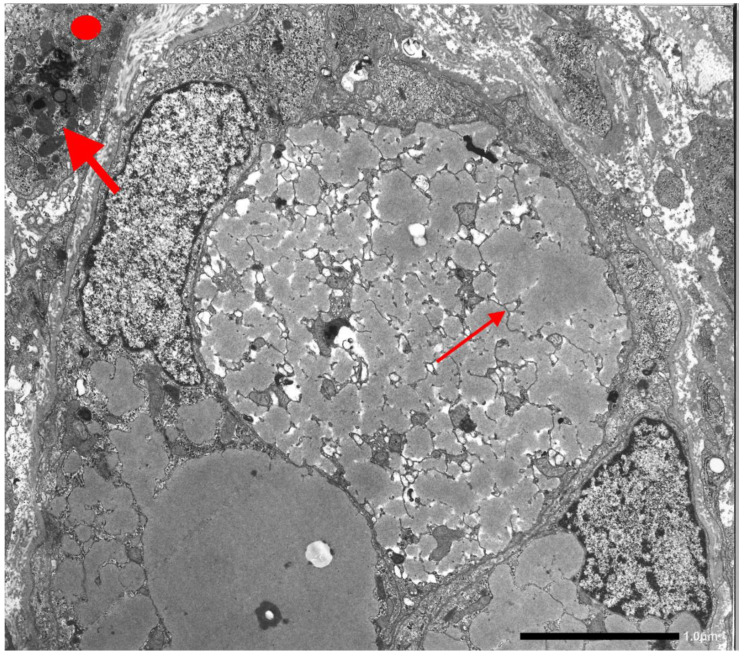
Pi ZZ liver. The cytoplasm of the hepatocytes is engulfed by Z AAT storage. Dilated cisternae of the RER contain AAT in the form of amorphous fluffy material and coalesce after disruptor of membranes and occupy the entire volume of the cell (thin arrow). Excess mitochondria (thick arrow) and glycogen rosettes (dot) indicate the periportal location of the cells (EM × 9200).

**Figure 13:**
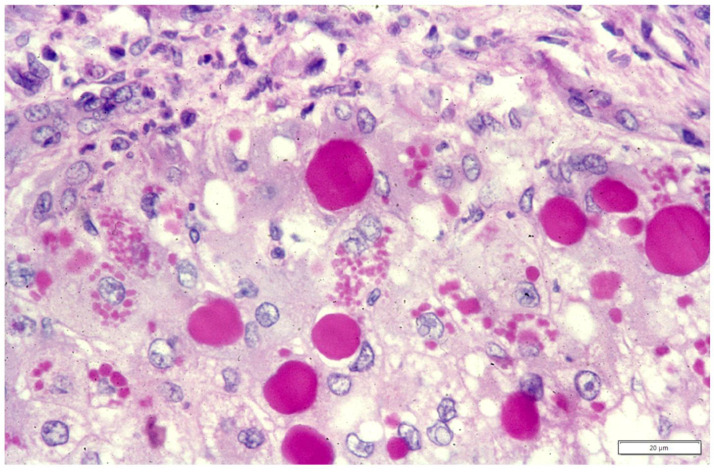
Liver tissue section from a Pi ZZ patient. Hepatocytes contain PAS–D inclusions of a variable size. A few occupy the entire cell volume (PAS–D × 40).

The retained protein within the RER causes cell engorgement and dysfunction. Ultrastructural damage to other cell organelles has been described [60]. Dilated cisternae of the RER may break or coalesce (Figure 12) into large inclusions that can occupy the entire cell volume (Figure 13).

**Figure 14:**
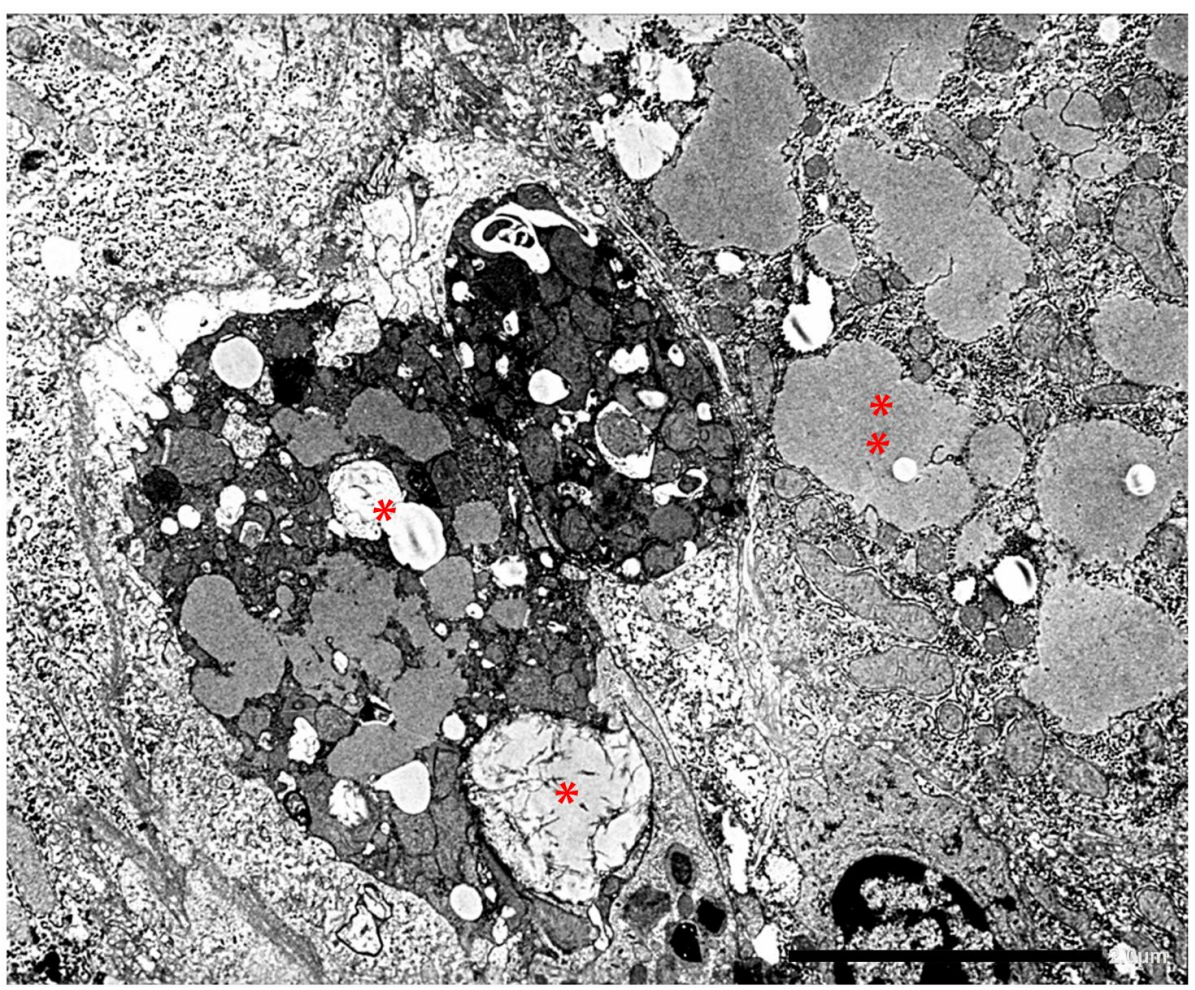
Pi ZZ cirrhotic liver. The electronmicrophotograph shows two adjacent hepatocytes with a dark appearance of the cytoplasm and severe organelle damage. The picture corresponds on light microscopy to apoptotic bodies. AAT-like material is present within dilated cisternae of the RER (**). In a few inclusions, the AAT appearance is dense, compact with remnants of membrane inside (*). Inflammatory cells and bundles of collagen type I are seen all around the apoptotic cells (EM × 6160). (Figure obtained from Callea F. PhD Thesis. Acco, Leuven 1983: 1–153.

The cell damage may progress to apoptosis (Figure 14), dropout of parenchymal liver cells, inflammation, fibrosis, and cirrhosis.

**Figure 15:**
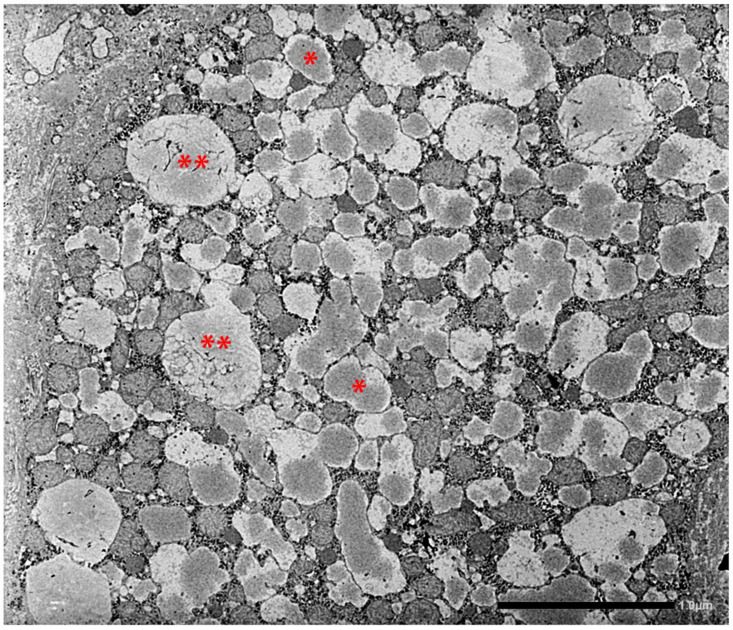
The EM-photograph from a Pi ZZ liver shows a hepatocyte whose ER cisternae are plenty of AAT-like material. AAT appears as amorphous fluffy material detached from the limiting membranes (*), or as a compact, dense material associated with disrupted ER membranes (**) (EM × 12.000). Figure obtained from Callea F. PhD Thesis. Acco, Leuven 1983: 1–153.

**Figure 16:**
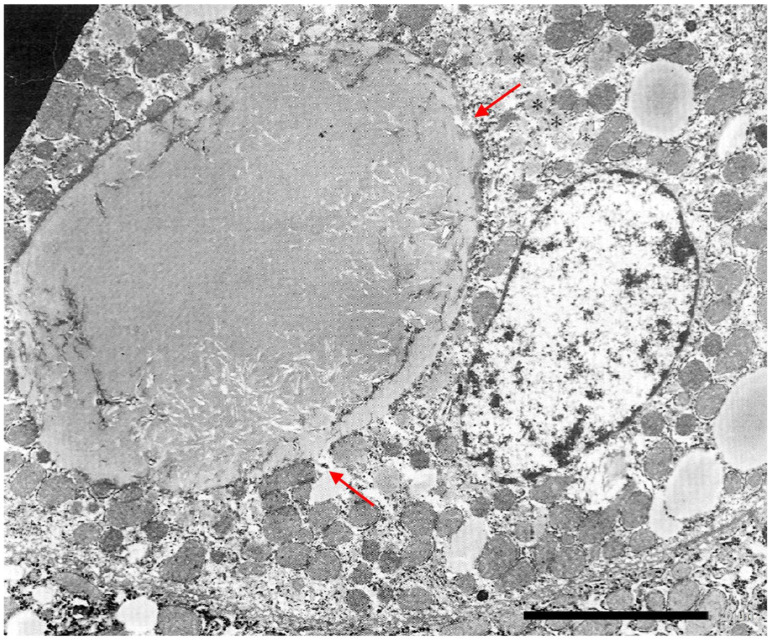
Pi ZZ liver specimen. The electronmicrophotograph shows a single large hepatocytic cytoplasmic inclusion with a compact, mummified appearance suggesting an intracellular stone. Broken ER membrabe are seen at the periphery (two separate arrows. Three small asterix (*) indicate small cisternae of RER containing AAT-like material with a flufy appearence (EM × 9200)). Figure obtained from Callea F. PhD Thesis, Acco, Leuven 1983: 1–153.

In addition to these features, we have observed that the intraluminal AAT, which usually appears as amorphous fluffy material detached from the ER membranes, can gradually become dense, compact (Figure 15), can entrap remnants of disrupted membranes and grow up to large dimensions, acquiring the appearance of a stone-like material (Figure 16).

The conventional fluffy appearance of ZAAT suggests either the potential for progression along the secretory pathway, albeit at a lower speed than normal protein, or the availability for proteolytic action by ER enzymes, retrotranslocation and degradation via autophagy or proteasome systems [61,62]. The EM fluffy appearance of ZAAT in the RER can represent the morphological counterpart of the Z polymers identified in circulation [63,64].

In contrast, the compact mummified ultrastructural appearance would reflect incompatibility either with progression along the secretory pathway or with degradation.

We had the opportunity to evaluate successive liver biopsies of Pi Z patients during the follow-up. In a few of them, we observed a significant decrease in the amounts of AAT storage. The observation was confirmed in multiple autopsy liver specimens, thus excluding the possibility of sampling errors [10]. The reduction of AAT storage was first observed by Starzl [65] in Pi Z patients following portacaval shunts. This clinical observation reflects an interesting phenomenon, especially in view of the ongoing research efforts to obtain the same results by drugs. The clinical and histopathological experience strongly suggests that portacaval shunt can reduce the storage by preventing further overload as a result of the diversion of the blood flow that deviates acute phase stimuli and pancreatic hepatotrophic products away from the liver [10,65].

With regard to the toxicity of the storage, the mineralization process occurring with the Mmalton AAT inclusions could be an additional cause of cell damage as calcium is a potential effector of cell death [58].

The pathogenesis of neonatal cholestasis in Pi ZZ newborns has been discussed in paragraph 1.2 of this article. A minority of cholestatic newborns develop childhood or infantile cirrhosis. The remaining patients may develop chronic hepatitis or cirrhosis in the adult age.

AATD deficiency significantly increases the risk of the developing hepatocellular carcinoma (HCC) [66]. An intriguing phenomenon has been described in Pi Z and Mmalton livers developing HCC. Tumor cells do not accumulate the mutant protein whilst non-tumorous hepatocytes are continuing to store the Z protein [67]. Interestingly, the same phenomenon occurs in HCC arising in Z transgenic mice [68]. This phenomenon was initially interpreted as an effect of retromutation in analogy to HCC arising in tyrosinemia type 1 [69]. A recent molecular study has shown that neoplastic hepatocytes carry the same mutation (Z or Mmalton) as the non-tumorous hepatocytes, thus suggesting a mechanism, possibly epigenetic, rather than a retromutation, to explain the lack of AAT expression [70].

The development of liver tumors in rodents, either spontaneous or experimentally induced, is characteristically age-related [71]. An increasing number of HCC has been reported in Z-transgenic mice [72,73]. In our series, we have observed more numerous HCC in older Pi Z mice than in wild type mice of the same age. In addition to HCC, we have found hepatic angiosarcoma and abdominal lymphoma never reported before [68].

Our observations appear to confirm the utility of transgenic Pi Z mice as a model for HCC development also in human. AAT globule depletion seems to be the common denominator and may reflect dedifferentiation, loss of function, re-expression of fetal markers and proliferation [68]. In contrast to other models [72,73,74,75], we did not observe inflammation, fibrosis or cirrhosis in our series of Pi Z mice [68].

### 2.5. Extrahepatic Manifestations of AATD

AATD deficiency has been reported in association with a number of diseases such as asthma, rheumatoid arthritis, anchylosing spondylitis, Weber-Christian panniculitis [8,18], Wegener granulomatosis [76], ANCA-positive systemic vasculitis [77], diabetes [78]. Most studies have been performed in small series on serum phenotyping [8]. However, for those carried out by AAT genotyping, like systemic vasculitis [79], however it is not definitely established whether the Z gene may predispose to the disease or act as a determinant of outcome [79].

The most important extrahepatic manifestation in AATD is pulmonary emphysema.

The development of emphysema is attributed to the insufficient protection of elastic alveolar tissue due to low serum AAT levels occurring only in Pi ZZ individuals. Heterozygous MZ individuals, due to the “Recruitment secretory block phenomenon” [27], are capable of raising AAT serum concentration up to protective levels. However, they become at risk if they are smokers. The cigarette smoke indeed causes direct oxidation of the single reactive methionine center [80,81] and inactivation of the inhibitory capacity of AAT that is by far the dominant antiprotease in the lower respiratory tract [82].

In the lungs of AATD individuals, AAT can be inactivated also by proteolytic cleavage and polymerization [83]. Polymerized ZAAT plays a distinct pro-inflammatory role by acting as a strong neutrophil chemoattractant [83]. By applying a 2C1 monoclonal antibody, specific for polymerized AAT, ZAAT polymers have been detected in alveolar macrophages of individuals with AATD as well as in smokers with normal AAT levels with or without chronic obstructive lung disease (COPD) [84]. According to the latter observation, the similarities in the pathophysiology of COPD in individuals with or without AATD, could help in the understanding of the mechanism of COPD [84].

The relationship between intra and extracellular AAT in both normal and pathological conditions is highlighted in diabetes. Carbohydrate intolerance and relative insulin deficiency in AATD was published as an abstract in the early seventies [85]. A role of the pancreas AAT in diabetes has been further discussed [86] and is becoming topic in view of the claimed prevalence of AATD and its comorbidities, including diabetes [87]. In addition, AAT treatment has shown a beneficial effect on diabetes and on beta cell regeneration in autoimmune diabetes [87].

## 3. Hereditary Hypofibrinogenemia with Hepatic Storage (HHHS)

Congenital fibrinogen disorders are caused by mutations that affect the synthesis and the secretion of fibrinogen, causing quantitative (afibrinogenemia or hypofibrinogenemia) or qualitative defects (dysfibrinogenemia). Mutations can occur in all three coding genes (FGA, FGB, FGG) [88]. Interestingly most cases of either a- or hypofibrinogenemia are not associated with fibrinogen accumulation in hepatocytes. Apparent exceptions are missense mutations or a single large deletion in the fibrinogen gamma chains [89,90] and in a single case report a missense mutation of the FGA gene [91] previously documented to be associated with dysfibrinogenemia but not with hypofibrinogenemia [92].

In this article, after shortly reviewing the synthesis and function of fibrinogen, we will focus on the variants of hypofibrinogenemia due to hepatic storage that are now classified as ERSD.

### 3.1. Fibrinogen Sources and Function

Fibrinogen is a highly pleiotropic protein involved in the final step of the coagulation cascade, in wound healing, inflammation and angiogenesis. Fibrinogen is primarily synthesized in hepatocytes from three homologous polypeptide chains (A-alpha, B-beta, gamma), encoded respectively by three genes, alpha FGA-, beta FGB-, gamma FGG-. The assembly of the three component chains into the six chain dimers occurs in the RER. After the terminal glycosylation in the Golgi complex, the mature molecule is exported into the blood [93,94,95]. Unassembled chains are not secreted and are degraded [93,94,95] by lysosomes or by the proteasome ubiquitin system [61,62].

Hepatocytes are the only recognized source for plasma fibrinogen. Megakaryocytes and platelets contain fibrinogen gamma chains within alpha granules. It has been historically controversial if the source of platelets’ fibrinogen is exogenous with incorporation by endocytosis from the circulation or if the source is a primary synthesis [96]. The presence of specific receptors for fibrinogen gamma chain on platelet membrane and the absence in bone marrow megakaryocytes or in circulating platelets of alpha and beta mRNA chains on PCR testing, suggest that the only possible mechanism responsible for the presence of fibrinogen in alpha granule platelets is endocytosis from plasma [96]. Of course, the final proof would come from RNA-PCR gamma chain studies

The platelet fibrinogen is believed to play a role in platelet aggregation and clot formation [96].

### 3.2. Hepatic Manifestations in HHHS

Mutations in any of the Fibrinogen gene (FGA, FGB, FGG) can result in hypofibrinogenemia [97]. However, only eight fibrinogen gamma chain mutations: Brescia (Gly384Arg), Aguadilla (Arg375Trp), Al du Pont Thr314Pro), Angers G316_Q350del), Beograd (Gly336Ser), Pisa (Asp316Asn), Ankara (His340Asp), Trabzon 8Thr371Ile) [89] and a single mutation on the A alpha chain (c.103C > T) [91] have been proven to result in hepatic storage and in a new disease, called hereditary hypofibrinogenemia with hepatic storage (HHHS) [1,15,16]. In all cases with hepatic storage, the gamma mutations were located at the end-to-end region of the globular D domain of the gamma chain and were hampering the D-dimer formation, causing aggregation of the protein within the RER [97,98]. In all other cases of congenital hypofibrinogenemia, the mutations were located far away from the crucial interface of the globular D-domain of the gamma monomers. The failure of correct polymerization leaves each monomeric gamma chain with exposed hydrophobic patches that give rise to undue interaction with lipids and with hydrophobic regions of APO-B-lipoprotein and other lipids [16,98,99].

For the single FGA mutation with hepatic storage [90], the information is limited. Lee et al. have used a bioinformatics tool without further characterization. The identified mutation (c.1036 > T) at codon 35 of the gene (FGA) was predicted to be probably damaging with a score of 1 by the Polymorphisms Phenotyping v2 (PolyPhen2) software.

Congenital hypofibrinogenemia can result from a variety of mutations in the three fibrinogen genes but does not produce clinically significant conditions. The mutations can affect transcription, mRNA processing, translation, post-translational processing, decreased proteolytic stability [97]. Most of the times, they are incidentally discovered during routine blood check or because of mild to moderate coagulation test abnormalities. Bleeding is rare and can occur after trauma or post-partum congenital hypofibrinogenemia patients do not have signs of liver disease and, as far we now, there is no single case in which the liver morphology has been studied. The only exception is the HHHS in which eight gamma chain mutations causes hypofibrinogenemia through the formation of intrahepatocytic inclusion bodies, similar to AATD [12,89]. All reported patients were diagnosed by histopathological examination of liver tissue specimens obtained for abnormal liver test of unknown origin. In the single case by Lee et al. [91], in which the mutation was located in the FGA chain, the liver biopsy was obtained after the incidental discovery of hypofibrinogenemia during a routine bloodwork as part of a preoperative evaluation. In that child hypofibrinogenemia was associated with abnormal transaminase levels [91].

The above-mentioned association between hereditary hypofibrinogenemia and APO-B-lipoproteinemia represents a new syndrome, characterized by a mutation in the fibrinogen gamma chain gene that provokes a secondary acquired obligatory retention of another protein that per se is normal [16,89,98,99].

**Figure 17:**
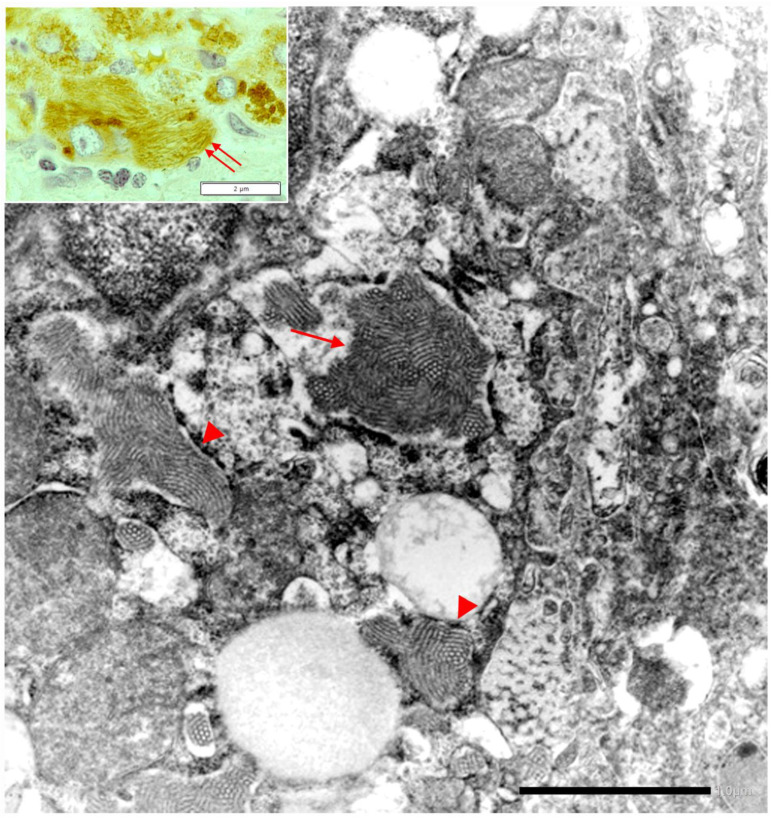
HHHS liver. The electronmicrophotograph shows a hepatocyte with dilated RER containing densely packed tubular structures arranged in curved bundles (fingerprint-like inclusions (thin arrow) or elongated fibers with a metameric array (arrowhead), corresponding to fibrinogen, reminiscent of extracellular fibrin (EM × 8000). On light microscopy the elongated fibers appear as acicular fiber glass-like inclusions (double arrows) (inset: fibrinogen immunostaining × 1200).

Fibrinogen storage in liver cells appears in the form of eosinophilic inclusions that are negative on PAS–D stain due to the low carbohydrate content, and strongly positive with specific anti-fibrinogen antibodies. The inclusions display a round/polygonal or acicular shape, the latter better visualized in immunostaining preparations (Figure 17, inset).

**Figure 18:**
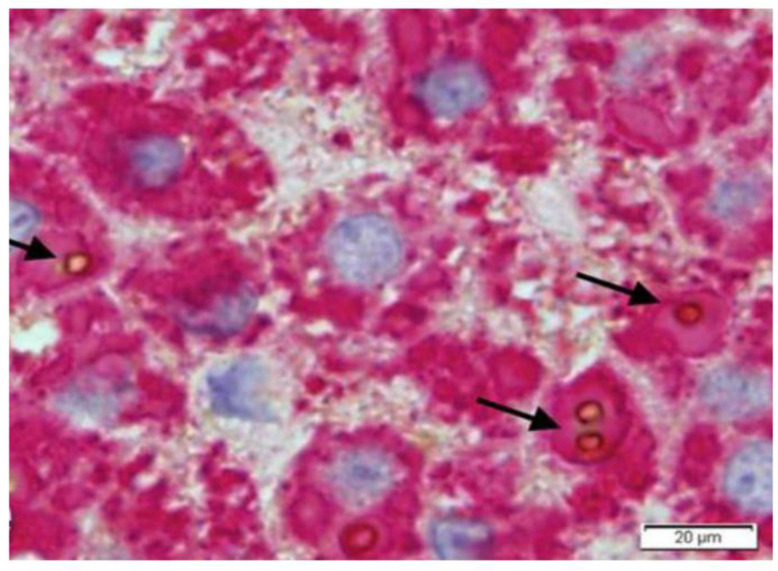
Liver tissue section from a patient with HHHS and hypo-APO-B- lipoproteinemia. All hepatocytes contain fibrinogen immunoreactive inclusions (red color). Round brown inclusions (arrows) represent lipid material inside fibrinogen inclusions. The periphery of lipids shows a positive immunoreaction to an anti-APO-B-lipoprotein antibody (double immunostaining, original magnification × 60). Figure obtained from Callea F et al. IJMS 2021; 22: 2899.

Apo-B-lipoprotein and other lipids are observed within fibrinogen inclusions (Figure 18) [99].

Under the EM, the stored fibrinogen presents as densely packed tubular structures arranged in curved bundles (fingerprint like) or in parallel metameric arrays (fiber glass like) (Figure 17) [12,13,14,15,16]. It is interesting and intriguing that this picture is vaguely reminiscent of the appearance of extracellular fibrin.

Like in AATD, the storage of fibrinogen in HHHS represents the elementary lesion of the liver pathology and is invariably associated with liver cell necrosis, hepatitis, fibrosis, and cirrhosis, mostly in early childhood.

The mechanism of liver damage in HHHS is poorly understood. In analogy with AATD, one may argue that the cell engorgement and the over-distension of RER cisternae could interfere with other organelles and cell function.

So far, in contrast to AATD, no single HHHS patient has developed HCC. However, it is important to underline that any poorly differentiated tumor, either in primary or metastatic sites, showing immunohistochemical positivity for fibrinogen can be diagnosed as HCC [10,67], due the fact that hepatocytes are the only fibrinogen synthesizing cells in the body [93,94,95].

### 3.3. Extrahepatic Manifestations in HHHS

The discovery of HHHS has allowed to identify the cause of a number of cases of liver cirrhosis that were previously considered cryptogenic, especially in children.

The new disease has been first described by pathologists in liver tissue specimens from patients with signs of chronic liver disease, unsuspected for suffering from fibrinogen abnormalities [15]. Initially, the disease has drawn the interest of pathologists and hepatologists, while hematologists have accepted with skepticism the new entity for two reasons: (i) it was the first time that hypofibrinogenemia resulted from hepatic storage, (ii) it was hardly believable that a severe hypofibrinogenemia could exist in a patient without overt coagulation problems or impairment of the main functions of fibrinogen [15].

In that respect, indeed, the first case report is paradigmatic. The patient was a 64-year-old female undergoing surgical cholecystectomy, a duodenal polypectomy and a surgical wedge biopsy because of unexpected liver cirrhosis [15]. After the histological diagnosis, fibrinogen plasma level was assessed and resulted to be very low (20 mg/dL). The patient had no bleeding and no delay in wound healing. On genotyping the mutation gamma284 Gly-Arg (Fibrinogen Brescia) was identified few years later [12].

Since then, seven more mutations on fibrinogen gamma chain with similar features have been found over the world [89]. In addition to fibrinogen Brescia, by using a standardized diagnostic methodology [1], we discovered two new mutations, Ankara [98] and Trabzon [89], and contributed to describe the first Italian [100], Japanese [101] Saudi Arabian [102], and Turkish [103] Aguadilla mutations. In total, 16 families with HHHS have been described. All patients undergoing a liver biopsy presented evidence of liver pathology. A minority of cases showed signs of mild bleeding (noise bruising) or hemorrhage in the post-partum period. A coagulation test showed mild alterations, thus leading to the statement that the low fibrinogen level and the mild derangement of coagulation test in HHHS are not at significant risk of bleeding. According to Asselta et al., coagulation problems in HHHS are less frequent than in hypo- or dysfibrinogenemia due to other chain mutations [104].

An intriguing phenomenon has been repeatedly reported with a few A-alpha chain mutations that result in systemic and especially renal amyloidosis [105,106]. This phenomenon appears to make a link between intracellular and extracellular conformational diseases and between two distant organs, liver and kidney. Interestingly, renal alpha-amyloidosis is successfully cured by liver transplantation [107].

## 4. Conclusions and Perspectives

AAT and fibrinogen can undergo molecule conformational abnormalities as a consequence of gene mutations and accumulate within the RER of hepatocytes. These features characterize ERSD [1] and conformational diseases [108], and are now included into the molecular organelle disease group [109] and into the chapter on metabolic errors and liver disease [110]. AAT and fibrinogen share also physiological properties that are relevant to the main purposes of this review: both circulating proteins are synthesized exclusively in the liver, are acute phase reactants and are highly pleiotropic proteins.

In addition, the two related diseases, AATD and HHHS, share cytotoxic liver cell damage and cirrhosis due to the storage. The latter is the hallmark of either diseases and represents the elementary lesion thus strengthening the genotype-phenotype correlation [16,58]. Liver disease occurs in both homozygous and heterozygous AATD. Heterozygous AATD (Pi MZ) cannot be considered carriers any longer as all of them have hepatic liver lesions [10,16,59] unavoidably leading to clinical liver diseases that cover the entire spectrum from chronic hepatitis to cirrhosis [111,112,113,114,115] and increases the risk of HCC development [111,113,115,116].

Interestingly, AATD and HHHS delineate a link between intracellular and extracellular conformational diseases and between liver and kidney with regard to renal amyloidosis that is cured by replacing the patient’s liver which, despite its normal structure and function, indirectly causes the renal pathology by producing an abnormal A alpha fibrinogen chain.

There are however several differences between the two proteins and the two diseases. In contrast to fibrinogen, AAT can be synthesized by several cell lines and tissues: macrophages, polymorphonuclear leukocytes, mast cells, Paneth cells, pancreatic islet, and sperm cells. As none of these cells contribute to the circulating AAT levels, it is argued that AAT in those cells plays a different role than the circulating one. This means that while AAT remains a preeminently secretory protein, it can be localized in lysosomes, condensing vacuoles, endocrine granules, and plasma membrane in cells other than hepatocytes. This intracellular AAT can be synthesized in the RER and proceed along the secretory pathway to the GERL area where it is diverted towards organelles, or it can be synthesized by free ribosomes with an addressing signal inserted during translation.

The intracellular AAT maintains the antiprotease activity and, within the cells, is presumably bound to proteases. The P-Pi complex would protect cell and organelles from self-digestion. The cleavage of AAT would allow the activation of pro-hormones into hormones as well as of proteolytic enzymes such as acrosin thus priming sperm cells to penetrate the zona pellucida of ovum.

The reason way in AATD, all hepatocytes can show accumulation of the mutant protein, while other cells appear to undergo storage rarely, is explained by the different role played by the protein. In the liver, AAT is per excellence an acute phase reactant protein and, as such, is recruited for a major synthesis and export to raise the serum level, whilst in other cell types which do not contribute to the circulating level, AAT plays an intracellular role and its synthesis takes place at a steady-state rate.

In conclusion, this paper has reviewed the results from original experimental studies for a Ph.D. thesis [10], and further developments published along a period of more than forty years. The initial rationale of those studies was based upon the assumption that the presence of a secretory protein inside the RER is a proof of an in situ primary synthesis. In that respect, AATD with the Pi MZ phenotype [10,27] has appeared as an experiment of nature and has finally led to forward the concept of ERSD, to discovery HHHS, and to clarify the intracellular role of AAT in extrahepatic locations.

The protean behavior of AAT is granting AAT as “a protein for all seasons”. Further studies are needed to demonstrate AAT mRNA in extrahepatic locations and to confirm the relationship between intra and extracellular protease inhibitor activity.

The striking similarity of intrahepatocytic aggregated fibrinogen and extrahepatic polymerized fibrin as well as the lack of overt hematological manifestations despite the very low fibrinogen level in HHHS, represent a challenge. The presumptive incompatibility with life of homozygous fibrinogen gamma chain mutations causing HHHS [17,94] requires extensive epidemiological studies and collection of additional HHHS families. It is hoped that the body of data collected over four decades of laboratory and clinical work, could serve as an useful background for future research. The final aim would be the treatment of ERSD with strategies aiming to reduce or prevent the hepatocytic/hepatotoxic storage of the genetically malformed proteins.

Useless and harmful could be the use of drugs, such as hormones or anabolizing steroids [27], as for any given amount of supplementary Z AAT-induced synthesis escaping into the serum, much more of it will be retained in the liver cells [10,27]. Obviously, these considerations cast doubts on the advantage of any procedure resulting in an increase of synthesis not accompanied by a parallel increase in the rate of secretion of the Z protein. Recent observations on small molecules predicted to bind Z AAT [117,118,119] or mutant fibrinogen at the interface of the aggregation sites, thus increasing degradation and inhibiting intracellular accumulation, appear to be promising and in line with the observations from the present study.

**Authors Contribution:** F.C. conducted the study design and wrote the manuscript; I.G. and P.F. performed the immunohistochemical and molecular analysis studies. All authors have read and agreed to the published version of the manuscript.
